# Beyond the Divide: Boundaries for Patterning and Stem Cell Regulation in Plants

**DOI:** 10.3389/fpls.2015.01052

**Published:** 2015-12-09

**Authors:** Shelley R. Hepworth, Véronique A. Pautot

**Affiliations:** ^1^Department of Biology, Institute of Biochemistry, Carleton University, OttawaON, Canada; ^2^Institut Jean-Pierre Bourgin, Institut National de la Recherche Agronomique, AgroParisTech, CNRS, Université Paris-SaclayVersailles, France

**Keywords:** meristem, lateral organ boundary, organ separation, inflorescence architecture, fruit patterning, flower patterning, abscission, dehiscence

## Abstract

The initiation of plant lateral organs from the shoot apical meristem (SAM) is closely associated with the formation of specialized domains of restricted growth known as the boundaries. These zones are required in separating the meristem from the growing primordia or adjacent organs but play a much broader role in regulating stem cell activity and shoot patterning. Studies have revealed a network of genes and hormone pathways that establish and maintain boundaries between the SAM and leaves. Recruitment of these pathways is shown to underlie a variety of processes during the reproductive phase including axillary meristems production, flower patterning, fruit development, and organ abscission. This review summarizes the role of conserved gene modules in patterning boundaries throughout the life cycle.

## Introduction

The shoot apical meristem (SAM) plays a crucial role in plant development as a continuous source of founder cells for provision of new leaves, shoots, and internodes throughout the life cycle. The SAM is organized into a central zone composed of slowly dividing stem cells, a peripheral zone where lateral organs initiate, and a rib zone that provides cells for internodes (Aichinger et al., 2012). The maintenance of meristems depends on the balance between two antagonistic activities: propagation of stem cells at the center of the meristem and the initiation of organs at the periphery. Boundaries are domains of restricted growth that maintain this balance by separating the meristem from the growing primordia and by forming an interface between organs (Žádníková and Simon, 2014). These interfaces play a critical role by influencing cell fate in adjacent tissues. The best-characterized boundary is the domain that separates leaves from the SAM during the vegetative phase. How principles governing the activity of this boundary apply to other developmental contexts is an important question. For example, boundaries in the leaf control shape and complexity whereas boundaries in the inflorescence have specialized functions such as axillary meristem (AM) production, fruit dehiscence, and organ abscission. Thus, many aspects of plant architecture are dependent on the boundary. In this review, we first describe the genetic control of boundaries during the vegetative phase, and then focus on elaboration of these pathways for specialized functions during the reproductive phase focusing on the model plant species *Arabidopsis thaliana* (*Arabidopsis*).

## Sam Initiation, Sam Maintenance And Cotyledon Separation

**FIGURE 1:**
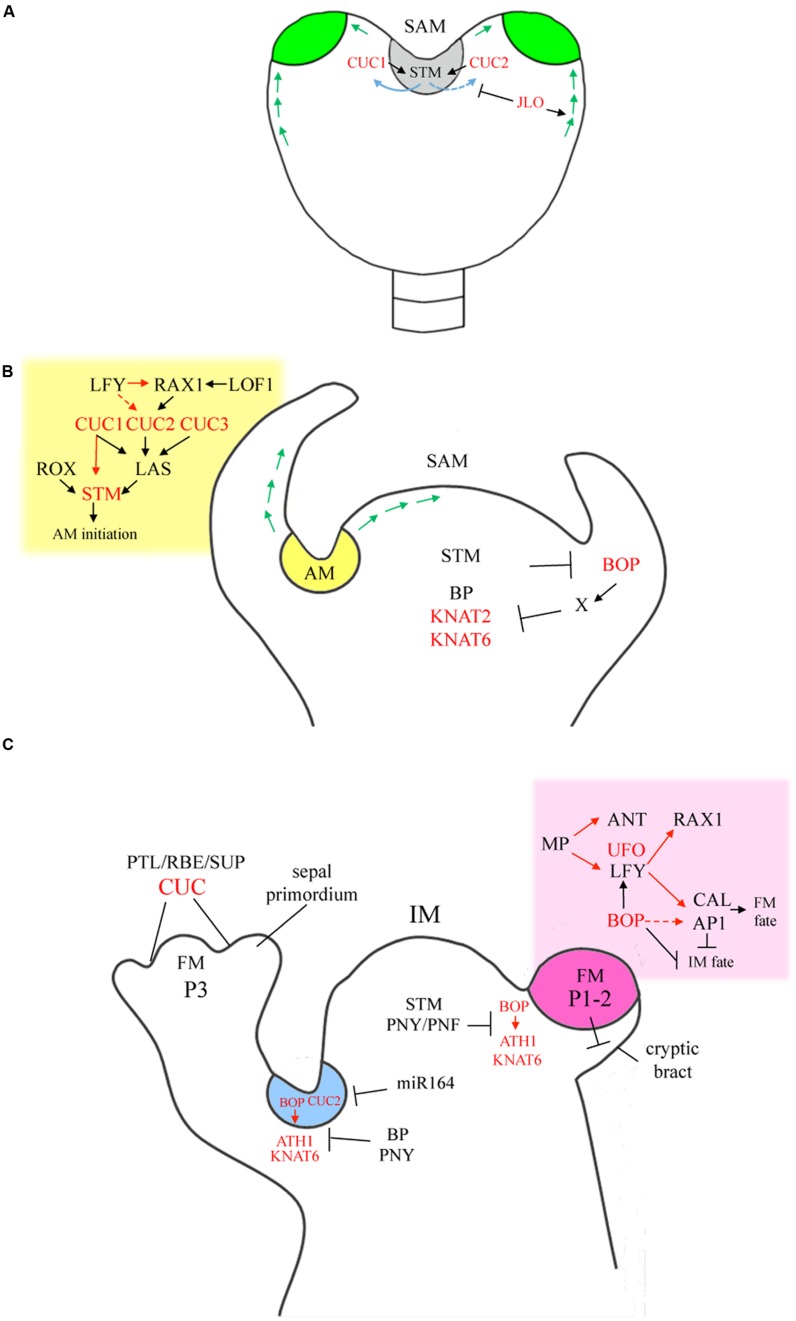
**Homologous boundary gene networks controlling biogenesis of shoot apical meristem (SAM), axillary meristems (AMs), and floral meristems (FMs). (A)** SAM initiation (gray). Early heart stage is shown. *CUC* genes are activated in a narrow band of auxin-depleted cells located between the presumptive cotyledons at globular stage. Activation of *CUC*s in this domain is partly dependent on chromatin remodeling ATPases and various other factors not depicted. Once activated, CUC1 and CUC2 are redundantly required for *STM* expression to form the presumptive SAM. STM in turn, directly maintains expression of *CUC1* and indirectly promotes *CUC2* and *CUC3* in establishing a feedback loop that ultimately restricts *CUC* expression to the axils of cotyledons. Reciprocally, *STM* expression is restricted to the SAM and slightly detected in boundaries. LBD family member JLO promotes *PIN1* expression required for formation of auxin maxima and represses *STM* and *KNOX* members to allow cotyledon outgrowth. Green arrows, direction of auxin flow. Green, auxin maxima at the cotyledon primordia. **(B)** SAM maintenance and AM formation (yellow). STM represses *BOP1/2* to maintain indeterminacy in the SAM. Conversely, BOP1/2 restrict *KNOX* expression in the proximal region of leaves to control patterning. Formation of an AM requires depletion of auxin from the leaf axil followed by a burst of CK. CUC1-3 are redundantly required for AM initiation functioning downstream of LFY and RAX1 to promote *LAS*. LOF1/2 contribute to *RAX1* promotion. CUC1, LAS, and ROX activities are required for sustained expression of *STM* and establishment of the AM. Green arrows, direction of auxin flow. **(C)** IM activity. PNY and PNF restrict *BOP1/2-ATH1-KNA*T6 expression to boundary domains flanking the IM essential for meristem maintenance and flowering. FMs (pink). FMs are AMs with determinate fate that form in the axil of leaves whose development are repressed (cryptic bract). Auxin responsive transcription factor MP directly activates *ANT* and *LFY* to initiate FM formation. LFY directly promotes the expression of *RAX1* and *AP1* and *CAL* whose products confer floral fate. BOPs facilitate establishment of FMs via promotion of *LFY* expression, activation of *AP1*, and repression of IM identity genes. UFO is a LFY co-activator also required for formation of boundaries in the flower. Later, CUC factors are required to separate floral organs and maintain boundaries between whorls in association with numerous stage-specific factors including PTL, RBE, and SUP required for localized repression of growth. Inflorescence architecture. BP and PNY are expressed in the stem cortex where they collectively promote internode elongation, stem differentiation, phyllotaxy, and pedicel angle by restricting boundary genes *BOP1/2* and downstream effectors *ATH1* and *KNAT6* to the pedicel axil (blue). Misexpression of these genes in the BP-PNY domain restricts growth, disrupts vascular patterning, and causes ectopic lignification. *CUC2* expression is restricted by *miR164* to the pedicel axil to maintain internode patterning. FM, floral meristem; IM, inflorescence meristem. P, primordia; stages as indicated. Red lettering, SAM-leaf boundary genes. Red arrows, direct regulation. Dashed line, putative interaction.

The NAM-ATAF-CUC (NAC)-type CUP-SHAPED COTYLEDON1 (CUC1), CUC2, and CUC3 transcription factors confer boundary identity in higher land plants (Maugarny et al., 2015). These factors initiate the SAM and establish boundaries in conjunction with SHOOT MERISTEMLESS (STM), a three-amino acid loop extension (TALE) class I KNOTTED1-like (KNOX) homeodomain protein [(**Figure 1A**) and (Hamant and Pautot, 2010; Hay and Tsiantis, 2010)]. CUC-STM forms a conserved module in development that was first identified in embryos (Aida et al., 1999; Takada et al., 2001; Aida et al., 2002). During the globular stage of embryogenesis, *CUC1* and *CUC2* genes are activated in a narrow band between the presumptive cotyledons (Aida et al., 1999; Takada et al., 2001), where auxin is depleted (Benková et al., 2003) based on positional cues provided by WUSCHEL-RELATED HOMEOBOX (WOX2) and WOX8/STIMPY-LIKE (Lie et al., 2012). CUC1/2 factors activate *STM* at late globular stage to initiate the meristem and separate the cotyledons (Aida et al., 1999). STM in turn maintains *CUC* expression (Aida et al., 1999). More recently, STM has also been identified as a direct regulator of *CUC1* (Spinelli et al., 2011). This pattern is reinforced by two SWI/SNF chromatin remodeling ATPase complexes BRAMHA (BRM) and SPLAYED (SYD) acting independently of STM: BRM is a positive regulator of all three *CUC* genes whereas SYD is required for *CUC2* expression (Kwon et al., 2006). By late torpedo stage, *STM* marks the central region of the meristem and is slightly detected in boundaries while expression of *CUC* genes is restricted to boundaries (Long and Barton, 1998; Aida et al., 1999). Double mutant analyses show that contributions of the different *CUC* genes are partially redundant. For example, *CUC1/2* are essential for meristem initiation while *CUC3* plays a more prominent role in organ separation (Vroemen et al., 2003) and AM production (Hibara et al., 2006). *CUC1* and *CUC2* transcripts are targeted by *microRNA164* (*miR164)* to restrict their expression domain while *CUC3* from a different subclade does not contain a *miR164* binding site (Laufs et al., 2004; Mallory et al., 2004).

Other three-amino acid loop extension (TALE) homeodomain transcription factors contribute redundantly with STM in SAM initiation and maintenance. The TALE superfamily is divided into KNOX and BELL classes, whose members function as heterodimers (Hamant and Pautot, 2010; Hay and Tsiantis, 2010). Formation of KNOX-BELL heterodimers regulates nuclear localization (Cole et al., 2006; Rutjens et al., 2009; Kim et al., 2013) and influences binding site selection (Smith et al., 2002). Within the *KNOX* subclass, *BREVIPEDICELLUS* (*BP*)/*KNOTTED1-LIKE FROM A. THALIANA1* (*KNAT1*) is expressed in the peripheral and rib zones of the SAM (Lincoln et al., 1994) whereas *KNAT6* is expressed in boundaries (Belles-Boix et al., 2006). Mutation in *BP* enhances only the meristem defect of weak *stm* mutants (Byrne et al., 2002) whereas *knat6* mutation also impairs cotyledon separation showing a specific role for *KNAT6* in boundaries (Belles-Boix et al., 2006). *KNAT2*, the fourth *KNOX* class I member is expressed at the base of the meristem and in lateral organ boundaries but its inactivation does not enhance the meristem defects of weak *stm* mutants (Byrne et al., 2002; Belles-Boix et al., 2006). *KNAT2* role in the SAM remains undetermined.

At least three BELL homeodomain proteins encoded by *PENNYWISE* (*PNY), POUND-FOOLISH* (*PNF*), and *ARABIDOPSIS THALIANA HOMEOBOX GENE1* (*ATH1*) interact with STM to maintain the SAM (Byrne et al., 2003; Kanrar et al., 2006; Rutjens et al., 2009). *PNY* is expressed in the central zone of the SAM (Smith et al., 2004); *PNF* is expressed in the central and rib zones of the SAM (Smith et al., 2004), whereas *ATH1* is more broadly expressed in the SAM, young leaves, and boundaries and is shown to control patterning in the basal region of shoot organs (Proveniers et al., 2007; Gómez-Mena and Sablowski, 2008). PNY and ATH1 contribute redundantly with STM in SAM initiation and maintenance (Byrne et al., 2003; Kanrar et al., 2006; Rutjens et al., 2009). PNY and PNF maintain the integrity of the central zone since the expression domain of *STM* is narrower in *pny pnf* double mutants (Ung et al., 2011). Meristem termination defects in this mutant are attributed to depletion of nuclear localized BELL-STM complexes (Rutjens et al., 2009), but recent data show that PNY and PNF negatively regulate lateral organ boundary genes including *ATH1* and *KNAT6* expression to maintain SAM function (Khan et al., 2015).

**FIGURE 2:**
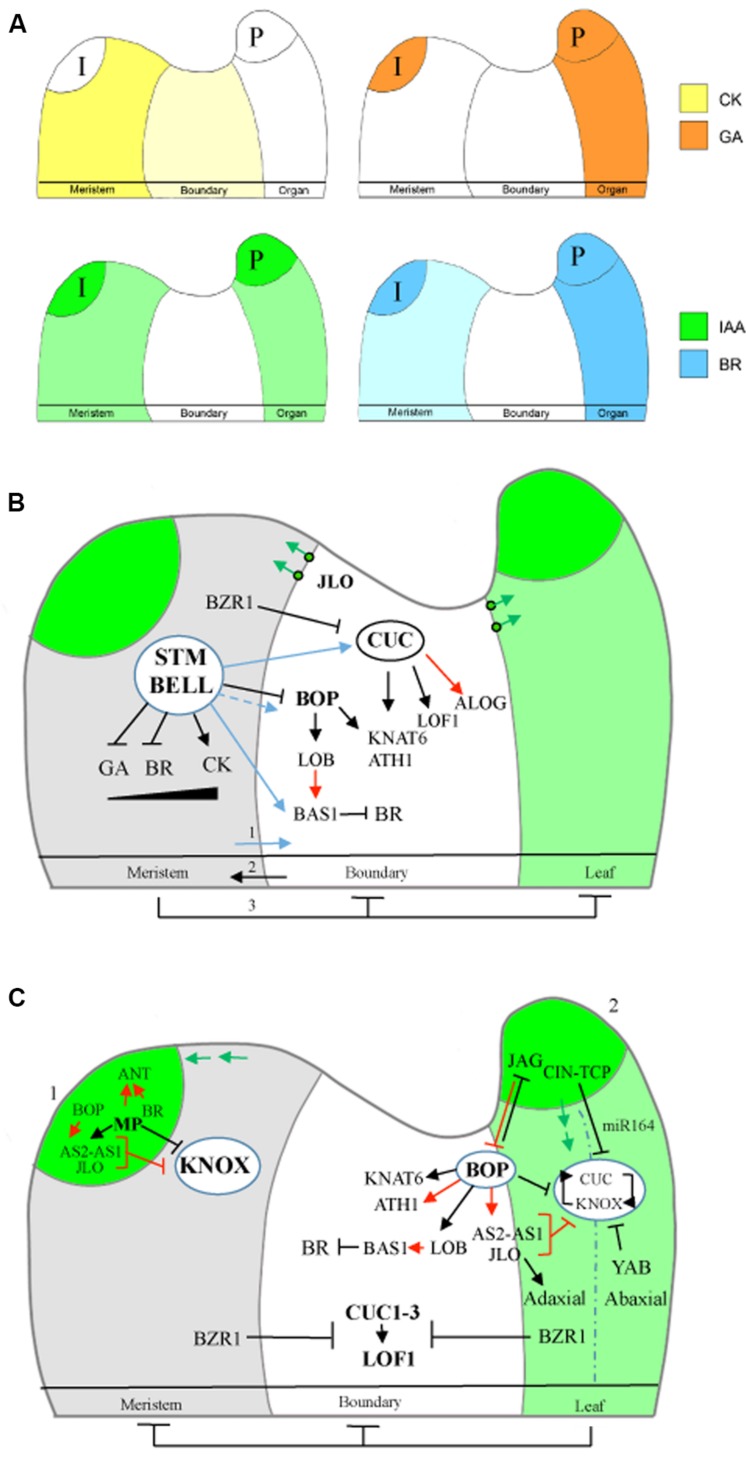
**Summary of hormone profiles and genetic interactions that maintain the leaf-SAM boundary. (A)** The shoot apex contains: the meristem, the initium, boundaries, and organ primordia. The predicted distribution of key hormones are summarized (see text for details). Meristem, high CK/IAA ratio and low GA/BR ratio promotes indeterminate growth. Primordia, high IAA activates BR and represses CK biosynthesis and GA increases to promote determinate growth. Boundary, depletion for growth-promoting hormones IAA, GA, BR, and CK inhibits cell division allowing separation of meristem-organ compartments. I, Initium; CK, cytokinin; BR, brassinosteroid; GA, gibberellin; and IAA, auxin. **(B)** Summary of gene networks at the meristem-boundary interface. Meristem, STM maintains indeterminate growth by promoting CK and repressing GA/BR accumulation (black gradient indicates hormone abundance). Auxin, shown in green, marks the site of primordia initiation and distal blade of emerging leaf. (1) KNOX proteins initialize the boundary through promotion of BR catabolic genes (*BAS1*) and boundary transcription genes including *CUC* and *BOP2* (blue arrows). CUC factors confer boundary identity required for activation of other classes of boundary regulators including BOP, KNAT-BELL, LOF1, ALOG, and LBD members that collectively restrict growth, modulate meristematic activity, and pattern the boundary. PIN1 auxin eﬄux carriers (green circles) are orientated facing outward such that auxin is drained away from the boundary. Green arrows indicate direction of auxin flow. (2) Boundary genes contribute to meristem maintenance (see text). (3) STM-BELL meristem factors preserve meristem integrity by restricting *BOP1/2* and *KNAT6* to boundaries. BR-activated transcription factor BZR1 represses *CUC/LOF1* in the meristem domain. **(C)** Summary of interactions at the leaf-boundary interface. (1) Polar auxin transport establishes auxin maxima in the peripheral zone where leaf initiation takes place. Auxin response factor MP initiates primordium formation by repressing *KNOX* genes, activating *ANT* members and leaf identity genes including AS1 and stimulating synthesis of BR where BZR1 binds to the *ANT* promoter as a positive regulator. Boundary genes *BOP1/2* and *JLO* expressed in the organ initial contribute to organ polarity and stable repression of *KNOX* genes. (2) Primordium outgrowth coincides with synthesis of auxin and repolarization of PIN transports toward the leaf base, which becomes a low IAA/BR domain. BOPs and JLO now restricted to the boundary reinforce this pattern in hormones via regulation of LOB and PIN1, respectively. CIN-TCPs and BZR1 in leaves maintain repression of *CUC/LOF1*. JAG in the distal blade represses *BOPs*. *BOPs* expressed in the proximal petiole domain of leaves maintain organ polarity and repress *KNOX* and *JAG* genes required for simple leaf shape indirectly in part via activation of *AS2*. YAB contributes to the repression of *KNOX* and *CUC* in the abaxial domain.

TALE transcription factors repress cellular differentiation in the meristem in part by regulating the abundance of hormones including gibberellins (GA), cytokinins (CK), and brassinosteroids (BR) (**Figures 2A,B**). A WUSCHEL-CLAVATA (WUS-CLV) feedback loop functions in parallel to keep the stem-cell niche constant in size (Schoof et al., 2000). A high CK: low GA ratio promotes meristem maintenance since high CK sustains cell division and low GA inhibits cell differentiation (Aichinger et al., 2012). Accordingly, meristem activity in *stm* mutants can be restored by elevating CK biosynthesis and inhibited by elevating GA abundance or signaling or by reducing CK content (Hay et al., 2002; Jasinski et al., 2005; Yanai et al., 2005). KNOX proteins raise CK levels by activating *ISOPENTENYL TRANSFERASE7* for CK biosynthesis (Jasinski et al., 2005; Yanai et al., 2005) and lower GA levels by directly inhibiting biosynthetic genes encoding GA20-oxidases (Sakamoto et al., 2001) and activating catabolic genes encoding GA2-oxidases (Bolduc and Hake, 2009). Genes encoding GA2-oxidase are expressed at the boundary between the SAM and leaves confining GA to leaves where growth is taking place (Jasinski et al., 2005; Bolduc and Hake, 2009). WUS contributes to this network by lowering the abundance of *ARABIDOPSIS* RESPONSE REGULATOR ARR7 and ARR15 (Leibfried et al., 2005) thus increasing sensitivity to CK in the central zone and promoting its own expression (Gordon et al., 2009). BR are a class of growth-promoting hormones recently shown to play a role in meristem maintenance (Tsuda et al., 2014). New work in rice and maize show that KNOX factors in the SAM maintain indeterminacy in part via direct activation of BR catabolism genes thereby downregulating BR signaling in the meristem. Inactivation of three rice orthologs of *Arabidopsis* catabolic gene *BAS1* (*PHYTOCHROME B ACTIVATION TAGGED SUPPRESSOR1*) results in the premature differentiation of meristematic cells (Tsuda et al., 2014).

## Boundary Formation

The boundary that separates the SAM from the primordia is a domain of restricted growth. This feature relies on depletion of auxin and BR from boundary cells thereby maintaining a low rate of growth relative to surrounding tissues (**Figures 2A,C**). Spatial regulation of polar auxin transporters establishes a minimum for auxin. This is accomplished in part by PIN-FORMED1 (PIN1) transporters oriented outwardly along the long axis of cells in the plane of the groove such that auxin is drained away from the boundary into the adjacent organ and meristem compartments (Heisler et al., 2010). Striking images of auxin depletion from the adaxial boundary domain of leaf primordia are observed using the auxin concentration sensor DII-Venus or auxin-responsive reporter gene *DR5:VENUS* (Wang et al., 2014a,b). ABC/PGP (ATP-binding cassette/*P*-glycoprotein) pumps are a secondary type of auxin transporter. ABC19 in this family depletes auxin from the boundary creating a low-auxin niche necessary for promotion of *CUC2* and *LOF2* expression (Zhao et al., 2013). An auxin minimum is essential in several boundary-related processes including AM formation (Tian et al., 2014; Wang et al., 2014a,b), differentiation of valve margins in the fruit (Sorefan et al., 2009), and timing of floral organ abscission (Estornell et al., 2013).

The boundary is also a minimum for BR (Bell et al., 2012; Gendron et al., 2012). The LATERAL ORGAN BOUNDARY DOMAIN (LBD) transcription factor LATERAL ORGAN BOUNDARIES (LOB) maintains low levels of BR to inhibit growth at boundaries. Ectopic expression of *LOB* reduces growth similar to BR defective mutants, while loss of *LOB* function causes overgrowth of the boundary region and organ fusion (Bell et al., 2012; Gendron et al., 2012). A feedback loop is required in establishing this pattern. Auxin-induced BR in the leaf activates *LOB1* (Chung et al., 2011) which in turn directs activation of cytochrome P450 gene *BAS1* to inhibit BR accumulation at the boundary (Bell et al., 2012). Two BTB-ankyrin transcriptional co-activators, BLADE-ON-PETIOLE1 (BOP1) and BOP2, reinforce this pattern by promoting *LOB* expression in the boundary domain (Ha et al., 2007). Fluorescent reporters show that BR-activated transcription factor BRASSINOZOLE-RESISTANT1 (BZR1) fails to accumulate in the nuclei of boundary cells thereby allowing expression of *CUC* genes which in turn repress growth at the boundary (Gendron et al., 2012).

Emerging data suggest that KNOX activity provides a positional cue in establishing the SAM-leaf boundary (Bolduc et al., 2012; Johnston et al., 2014; Tsuda et al., 2014). Several mechanisms are identified. One study shows that rice *KNOX* gene *Oryza sativa homeobox1* (*OSH1*) expressed in the meristem and base of emerging leaves facilitates SAM function and boundary formation by lowering BR abundance (Tsuda et al., 2014). Transcriptomic studies in maize focusing on the blade-sheath boundary of leaves further reveal that *CUCs*, *TALEs*, and *BOPs* are downstream targets of KNOTTED1 (KN1) under positive regulation (Bolduc et al., 2012; Johnston et al., 2014). Grasses have a blade-sheath boundary containing hinge-like auricles that control leaf angle and a fringe of epidermal tissue called the ligule whose formation is under the control of boundary genes. Barley *UNICULME4* is a *BOP* homolog required for ligule outgrowth (Tavakol et al., 2015). Maize genes required for ligule development include *LIGULELESS2 (LG2)* which encodes a TGA bZIP factor (Walsh et al., 1998), *LG3/LG4* which are closely related genes to *Arabidopsis KNAT2* and *KNAT6*; and *KNOTTED1/ROUGH SHEATH1* homologs of *STM* or *BP* (Bolduc et al., 2012; Johnston et al., 2014). Pre-ligule tissue is enriched for homologs of *CUC2*, *BOP1/2*, *KNAT6*, and two *BELL* genes, whose loci in several cases are bound directly by KN1 as identified through chromatin immunoprecipitation assays (Bolduc et al., 2012; Johnston et al., 2014). These data support a model in which KNOX accumulation at the base of the leaf primordia and auxin accumulation in the distal portion of the primordia provide opposing positional cues in demarcating the blade-sheath boundary (Bolduc et al., 2012; Johnston et al., 2014). *Arabidopsis* studies showing that STM directly activates *CUC1* and indirectly promotes *CUC2*, *CUC3*, and *BOP2* expression (Spinelli et al., 2011) support this model.

Genetic studies show that *CUC* genes play a central role in maintaining growth repression in boundaries. Inactivation of any two *CUC*s leads to ectopic growth at cotyledon boundaries causing fusion along their margins (Aida et al., 1997; Vroemen et al., 2003). GROWTH-REGULATING FACTORS (GRFs) which act as broad regulators of cell proliferation function synergistically with CUCs in this role. Leaf fusion defects in *grf1 grf2 grf3* triple mutants are dramatically enhanced by inactivation of *GRF4* or *CUC* genes resulting in cup-shaped cotyledons and embryos that lack a functional SAM (Lee et al., 2015). Two *ALOG* (*Arabidopsis LSH1* and *Oryza G1*) family members *ORGAN BOUNDARY1/LIGHT-DEPENDENT SHORT HYPOCOTYL* (*OBO1/LSH3*) and *OBO4/LSH4* are direct targets of CUC1 and thought to repress differentiation of boundary cells (Cho and Zambryski, 2011; Takeda et al., 2011). Another regulator is the MYB transcription factor LATERAL ORGAN FUSION1 (LOF1), which promotes organ separation and meristem maintenance. Inactivation of *LOF1* enhances *stm-10* meristem termination and organ fusion defects (Lee et al., 2009).

## Organ Initiation

One of the earliest steps in initiation of lateral organs is down-regulation of *STM* at sites of auxin maxima in the peripheral zone of the meristem. Boundary and leaf identity genes are transiently expressed in the same compartment undergoing differentiation. Various studies show that organ initiation and boundary formation are interconnected processes (Heisler et al., 2005; Besnard et al., 2011). Auxin in the distal portion of the primordia controls the localization of boundary genes ultimately restricting their expression to the base of the emerging leaf (**Figures 2A,C**). In brief, cotyledons and leaves are initiated at auxin maxima generated by polar auxin transport. Polar auxin distribution is dependent on a family of eﬄux carriers including PIN1 whose membrane localization is controlled by the serine/threonine kinase PINOID. Threshold levels of auxin trigger activation of the auxin-responsive transcription factor MONOPTEROS (MP), which down-regulates *STM* and activates *AINTEGUMENTA (ANT)*, *ANT-like (AIL*), and *ASYMMETRIC LEAVES1 (AS1)* genes to initiate leaf development (Long and Barton, 1998; Besnard et al., 2011; Yamaguchi et al., 2013). As the primordium emerges, PIN1 polarity reverses to generate new auxin peaks coinciding with a narrow band of cells marked by *CUC* expression (Heisler et al., 2005). Mutations in *PIN1*, *PID*, or *MP* that disrupt auxin transport or signaling lead to expansion of *STM* and *CUC* expression to the periphery where they suppress cotyledon outgrowth (Aida et al., 2002; Furutani et al., 2004; Schuetz et al., 2008). Proper distribution of auxin in forming this pattern is dependent on SEUSS and SEUSS-like components of the LEUNIG repressor complex although the mechanism is still unknown (Lee et al., 2014). Auxin in the leaf initial further alters the balance of hormones to favor growth and determinacy. In particular, auxin stimulates BR (Chung et al., 2011) and GA synthesis (Frigerio et al., 2006) and represses CK production (Nordstrom et al., 2004; Besnard et al., 2011). Primordium outgrowth also depends on physical changes in cell wall stiffness (Besnard et al., 2011; Peaucelle et al., 2011). Auxin stimulates the active transport of protons into the extracellular space required in activating enzymes that relax the cell wall and promotes the transcription of remodeling factors including expansions, pectin methylesterase, and hydrolases (Besnard et al., 2011; Peaucelle et al., 2011). These changes are coupled with a shift toward growth isotropy, which facilitates organ outgrowth (Sassi et al., 2014).

## Leaf Differentiation

Leaf differentiation requires the maintenance of *KNOX* repression and the restriction of *CUC2/3* expression along the leaf margin. *KNOX* repression is accomplished by an interacting network of leaf and boundary factors (**Figure 2C**). A key player in this network is the MYB transcription factor AS1, which acts in a trimeric complex with the LBD transcription factors AS2 and JAGGED LATERAL ORGANS (JLO) (Guo et al., 2008; Rast and Simon, 2012). AS1 and AS2 bind to distinct sites in the *BP* and *KNAT2* promoter where they interact through looping to induce silencing via recruitment of the histone chaperone HIRA and Polycomb-repressive complex2 (Guo et al., 2008; Lodha et al., 2013). *STM* is also a target of PRC but how this complex is recruited to the promoter is unknown (Lodha et al., 2013). *JLO* is transiently expressed at sites of organ initiation and resolves to the leaf-meristem boundary during outgrowth. Loss-of-function mutations in *JLO* impair organ outgrowth and enhance the margin patterning defects of *as2* mutants. This phenotype is caused in part by ectopic expression of *BP* and *STM* at the base of leaf primordia combined with defects in auxin distribution (Rast and Simon, 2012). JLO promotes *PIN* expression for auxin build-up at organ initiation sites and later for auxin eﬄux from the boundary (Bureau et al., 2010; Rast and Simon, 2012; Žádníková and Simon, 2014).

BOP1/2 activity in organ initials partially overlaps with *JLO* and likewise resolves to the boundary of emerging leaves and petiole domains during outgrowth (Ha et al., 2004; Hepworth et al., 2005; Norberg et al., 2005; Borghi et al., 2007). BOP1/2 have a dual function. They repress genes that confer meristem cell fate and induce genes that promote lateral organ fate and polarity (Ha et al., 2007). *BOP1/2* transcripts are first detected in the boundaries of torpedo stage embryos consistent with a function downstream or in parallel with CUCs (Ha et al., 2004). STM represses *BOP1/2* to maintain indeterminacy and conversely, BOP1/2 restrict *KNOX* expression to pattern the leaf (Jun et al., 2010). A prolonged phase of morphogenetic competence in *bop1 bop2* petioles coupled with *KNOX* reactivation results in initiation of ectopic leaflets reminiscent of development in a compound leaf (Ha et al., 2003, 2007; Khan et al., 2014). BOP1 binds directly to the promoter of *AS2* likely recruited by a TGA factor (Jun et al., 2010). Synergistic enhancement of meristematic activity in *bop1 bop2 as1* and *bop1 bop2 as2* petioles shows that BOP1/2 repression of *KNOX* genes is not entirely via AS1–AS2 and is likely indirect. Leaf patterning defects in *bop1 bop2* are also attributed to misexpression of abaxial/adaxial organ polarity determinants and the C2H2 zinc finger transcription factor JAGGED (JAG) which promotes cell proliferation (Norberg et al., 2005; Ha et al., 2007, 2010). JAG is normally restricted to the distal blade where it represses *BOP2* to allow extension of the leaf margin (Schiessl et al., 2014).

CINCINNATA (CIN)-like TEOSINTE BRANCHED1/CYCLOIDEA/PCF (TCP) factors are another class that contribute to negative regulation of *CUC* and *KNOX* activity in leaves to promote organ outgrowth and simple leaf shape (Koyama et al., 2007, 2010). Consistent with this view, *CIN-TCPs* are predominantly expressed in leaves and depleted from the boundary (Tian et al., 2014). Several mechanisms are identified. First, TCP3 directly promotes *miR164*, which targets *CUC1* and *CUC2* transcripts for cleavage. Second, TCP3 directly promotes *AS1* whose product represses *CUC3* and *KNOX* expression (Koyama et al., 2010). In addition, TCP3 targets auxin inducible genes that repress SAM function and cause cotyledon fusion when overexpressed (Koyama et al., 2010). Lastly, TCP4 binds to CUC2 and inhibits its activity by blocking the formation of homo-dimers and hetero-dimers with CUC3. TCP4 also impairs CUC3 transactivation ability (Rubio-Somoza et al., 2014).

## Leaf Shape

Variations in the KNOX-PIN-CUC module play a central role in controlling leaf shape and complexity. The leaf margin, due to its meristematic feature, is particularly sensitive to alterations in this module. In simple leaf species such as *Arabidopsis, CUC2*, and *CUC3* expression is restricted to the sinus of serrations along the leaf margin while *CUC1* expression is not detected (Nikovics et al., 2006; Hasson et al., 2011). The balance between *CUC2* and *miRNA164* transcripts controls the degree of leaf serration (Nikovics et al., 2006). CUC3 contributes to leaf shape at a later stage (Hasson et al., 2011). Similar to the primordia initiation, the formation of serrations depends on auxin. To explain how serrations form on a leaf margin, Bilsborough et al. (2011) proposed a model in which two feedback loops work in concert. In the first loop, PIN1 convergence in the leaf margin generates an auxin maximum, reinforced by auxin feedback on PIN1. In the second loop, CUC2 acts non-cell autonomously to promote growth through the generation of PIN1-dependent auxin maxima and contributes to tooth outgrowth (Kawamura et al., 2010). Auxin in turn downregulates *CUC2* restricting expression to regions between serrations where growth is repressed.

The rachis of a compound leaf is likewise sensitive to alterations in *KNOX-PIN-CUC* expression. While simple leaves have a single undivided blade in which *KNOX* repression is continuous, compound leaves have a divided blade consisting of pairs of leaflets attached to a central rachis. This morphology is associated with an extended primary morphogenesis phase during which reactivation of *KNOX* genes begins the cycle by promoting auxin accumulation thereby directing leaflet initiation on the rachis (Di Giacomo et al., 2013). Down-regulation of tomato *BOPa* (one of three homologs) further enhances leaf complexity by extending the window for rachis responsiveness to auxin (Ichihashi et al., 2014). BOPa fulfills this function in part by forming a complex with LIGHT-DEPENDENT SHORT HYPOCOTYL3b (an ALOG family member) that represses tomato *KNATM* encoded by *KD1/PETROSELINUM* to modulate KNOX activity (Ichihashi et al., 2014). KNATM, a mini-KNOX lacking the homeodomain, modulates KNOX-BELL activity by competing for BELL binding partners (Kimura et al., 2008; Magnani and Hake, 2008). Analysis of the maize KN1 cistrome confirms that a majority of directly regulated genes are involved in auxin signaling, biosynthesis, and transport including PIN1 (Bolduc et al., 2012). Several legume species such as *Medicago truncatula* and pea use orthologs of *Arabidopsis LEAFY (LFY)* as an alternate source of meristem activity but remain reliant on CUC2 function for creation of auxin peaks required in leaflet initiation (Nikovics et al., 2006; Blein et al., 2008; Efroni et al., 2010). These data illustrate that variations in the KNOX-PIN-CUC module cause diversity in leaf patterning. Recruitment of this same module at later stages governs AM formation, gynoecium, and ovule development (Ishida et al., 2000; Hibara et al., 2006; Scofield et al., 2007; Raman et al., 2008).

## New Frontiers

In the next part of the review, we examine the role of boundary genes during the reproductive phase. Boundaries in the inflorescence determine plant architecture through the separation of organs and the distribution of flowers on the stem but also constitute a source of AM for production of branches and flowers. Boundaries are also sites where abscission and dehiscence take place. A number of genes are recurrently expressed in these boundaries including *KNOX-BELL*, *BOP*, and *CUC/miR164* regulators. Similar to the role of TALE factors in the SAM, PNY and PNF preserve meristem integrity essential for flowering by excluding *BOP1/2/KNAT6-ATH1* from the meristem. Inflorescence architecture is likewise controlled by restricting *CUC2* and *BOP1/2-KNAT6/ATH1* to boundaries at the base of floral shoots. CUC-STM factors play a conserved role in formation of new meristems, including AMs that give rise to lateral branches and flowers and meristematic tissues internal to the fruit. CUC factors also play a critical role in separation of floral organs and ovules in developing flowers. TALE factors including BP and PNY preserve formation of meristematic replum tissue in fruits whereas BOP1/2-KNAT6/2, which are expressed in adjacent valve margin boundary tissues potentially contribute to dehiscence. This same network of TALE and BOP factors regulates abscission. How these conserved modules are integrated during reproductive development is now discussed.

## Axillary Meristems

The boundary located between the stem and the leaf base constitutes a source of AMs, which can remain dormant or produce secondary inflorescences and flowers (**Figure 1B**). The specification and the development of AMs involves numerous transcription factors and is modulated by hormones such as auxin, CK, BR, and strigolactones (Janssen et al., 2014). Other hormones may also be involved based on specific patterns of enrichment for abscisic acid and ethylene or depletion of jasmonic acid (JA) responsive genes in the boundary domain (Tian et al., 2014). Similar to the SAM, formation of AMs requires CUC-STM factors whose activity at the boundary is dependent on auxin and CK.

Recent studies in *Arabidopsis* and tomato show that the establishment of a stem cell niche in leaf axils requires auxin depletion followed by pulse of CK (Wang et al., 2014a,b). Manipulation of the auxin gradient using chemical inhibitors of auxin transport or mutations in auxin transport machinery including PIN1 or PID showed that disruption of auxin minima strongly inhibits AM initiation (Wang et al., 2014a,b). STM, which is a marker of AMs in the mature leaf axil (Grbic and Bleecker, 2000; Long and Barton, 2000), fails to accumulate in a strong *pid-9* mutant indicating that its expression is dependent on an auxin minimum (Wang et al., 2014a). CK perception and signaling is enhanced in leaf axils prior to AM initiation and the TCS::GFP (two-component output sensor) synthetic reporter used to visualize CK response indicates that a pulse of CK follows the auxin minimum and is required to stimulate AM production (Wang et al., 2014b). STM may contribute to this pulse based on its CK promoting activity in the SAM (Jasinski et al., 2005; Yanai et al., 2005). *Arabidopsis* mutants affected in CK perception (histidine kinase receptor mutants) or CK signaling (ARR-B type transcription factor mutants) show reduced AM production whereas overproduction of CK restores AM initiation in a *rax1* mutant (Wang et al., 2014b).

REGULATOR OF AXILLARY MERISTEM1 (RAX1) is a MYB transcription factor that specifies AMs in redundancy with RAX2 and RAX3 (Keller et al., 2006; Muller et al., 2006). One regulator of RAX1 is the MYB transcription factor LOF1 which also functions to promote AM and organ separation (Lee et al., 2009). RAX1 acts through CUC2 and is required in conjunction with CUC3 and the GRAS-domain protein LATERAL SUPPRESSOR (LAS) to maintain *STM* expression in AMs. RAX1 maintains the boundary zone through the repression of GA similarly to STM in the SAM (Keller et al., 2006; Muller et al., 2006). *LAS* and *RAX1* promote AMs via the bHLH transcription factor REGULATOR OF AM FORMATION (ROX; Yang et al., 2012). LFY, which is involved in flower specification has recently been shown to promote AM proliferation through its direct target *RAX1* and potentially others (Chahtane et al., 2013). Redundant pathways mask this role as mutations in *LFY* combined with mutations affecting various pathways including meristem, auxin signaling, floral transition and patterning, or boundary genes such *BOP1/2* show defects in AM formation (Chahtane et al., 2013). LFY further contributes to meristem emergence via CK signaling potentially through its interaction with WUS. The negative regulator of CK signaling, *ARABIDOPSIS RESPONSE REGULATOR7* (*ARR7*), was found to be up-regulated in *lfy-12* inflorescences, and LFY interacts directly with the *ARR7* promoter (Moyroud et al., 2011; Winter et al., 2011; Chahtane et al., 2013). LFY may also act through *CUC2* and the auxin signaling pathway as genes from this pathway are bound by LFY (Moyroud et al., 2011; Winter et al., 2011; Yamaguchi et al., 2013). Thus, meristem emergence results from the convergence of LFY and LAS pathways.

Genetic studies show that *CUC* genes contribute redundantly and differently to AM initiation and boundary maintenance with *CUC3* playing a prominent role (Hibara et al., 2006). Inactivation of *CUC1* and *CUC2* has no effect on AM initiation and an effect in *cuc2* mutants is seen only in the absence of *CUC3* (Hibara et al., 2006). Further studies show a contribution of CUC1/2 to AMs. Overexpression of *miR164* dramatically reduces the initiation of AMs in the *cuc3-2* mutant, and reciprocally, *miR164-*resistant versions of *CUC1* and *CUC2* form extra accessory side shoots (Raman et al., 2008). Key downstream targets of the CUC pathway include *STM* and *LAS* required in AM establishment (Raman et al., 2008), with *LAS* being a direct target of CUC2 (Tian et al., 2014). *STM* expression is missing in the adaxial boundary domain of *cuc3-2* mutants and *LAS* expression is reduced in *miR164* overexpressing lines, which diminishes *STM* expression (Greb et al., 2003; Raman et al., 2008).

Translatome analysis of *LAS*-expressing boundary and *AS1*-expressing leaf primordia cells coupled with genome-scale mapping of transcription factor binding sites reveals that CUC2 and LAS are regulatory hubs for AM initiation (Tian et al., 2014). This work identifies the auxin-induced APETALA2 domain transcription factor DORNRÖSCHEN as a direct activator of *CUC2.* This work also identifies SQUAMOSA PROMOTER BINDING PROTEIN-LIKE9 and 15 as repressors of *LAS* and *CUC2* that regulate AM initiation likely in response to environmental signals (Tian et al., 2014).

Separation of axillary shoots from subtending leaves and formation of accessory side shoots requires LOF1 and LOF2 acting downstream of *CUC* genes (Lee et al., 2009; Gendron et al., 2012). *Lof1* defects in organ separation are enhanced by mutation of the closely related *LOF2* whose expression is more widespread but dependent on *LOF1* at the boundary (Lee et al., 2009). *STM* expression in AMs is reduced in *lof1 lof2* double mutants (Lee et al., 2009). Overexpression of a cysteine-rich signaling peptide TAXIMIN1 mimics the phenotype of *lof1 lof2* double mutants (Colling et al., 2015). Interestingly, this phenotype is not due to a reduction of *LOF1/2* or other boundary transcripts suggesting an independent mechanism (Colling et al., 2015). A peptide signaling cascade has not been previously linked to formation of boundaries in plants.

Studies in other species show this hierarchy to be highly conserved (Janssen et al., 2014). Tomato *GOBLET (GOB)* encodes a NAC-domain transcription factor similar to CUC2 (Berger et al., 2009); LAS is an ortholog of *LAS* (Schumacher et al., 1999; Greb et al., 2003); and *BLIND* is an ortholog of *RAX1* (Schmitz et al., 2002). Remarkably these same genes are regulators of leaf complexity. Homologous genes have also been identified in cereals as regulators of tillering and panicle architecture. LAX PANICLE1 in rice and BARREN STALK in maize encode bHLH proteins orthologous to ROX in *Arabidopsis*. Mutants in these genes show a reduction in panicle branches and spikelets and fail to form AMs during the vegetative phase resulting in a reduction in tillers (Komatsu et al., 2003; Gallavotti et al., 2004; Oikawa and Kyozuka, 2009). These proteins sustain early proliferation of the AM by forming a boundary between the meristem and axillary bud (Oikawa and Kyozuka, 2009; Yang et al., 2012). Barley *CUL4* is a BOP homolog required for tiller formation (Tavakol et al., 2015). BOP1/2 are required for production of various determinate axillary shoots including stipules, nectaries, and flowers in dicots (Khan et al., 2014). *BOP* expression is down-regulated at an early stage of indeterminate IM formation and moves to the boundary between the meristem and AM demonstrating a transient role similar to ROX (Xu et al., 2010; Yang et al., 2012). The contribution of CUL4 in AMs production suggests a partial conservation of BOP function in monocots and dicots.

## Inflorescence Architecture

The maintenance of boundaries during stem growth is critical in preserving plant architecture. Ectopic expression of boundary genes prevents the elongation and proper differentiation of stem internodes resulting in aberrant phyllotaxy. This is illustrated by clustering of flowers on the stems of plants expressing a *miR164*-resistant version of *CUC2* (Peaucelle et al., 2007). The restriction of *CUC2* expression to the floral stem axil by *miR164* in the IM is required to maintain the boundary between the pedicel and the stem (**Figure 1C**). The TALE transcription factors BP and PNY constitute another set of architecture determinants (**Figure 1C**). These factors are required to maintain internode patterning during stem growth and radial patterning in both primary and secondary phases of stem development (Smith and Hake, 2003). BP and PNY are expressed in the stem cortex and adjacent vascular tissues and form a boundary between the IM and lateral organs (Smith and Hake, 2003). Mutations in *BP* lead to short compact internodes, downward pointing siliques, and precocious outgrowth of paraclades (Douglas et al., 2002; Venglat et al., 2002). Vascular bundles in *bp* mutants are often irregular in size and/or spacing. Bundles tend to be underdeveloped with xylem elements reduced or lacking in lignin (Smith and Hake, 2003). Mutations in *PNY* cause shortened internodes and clusters of flowers on stems and partial loss of apical dominance (Byrne et al., 2003; Smith and Hake, 2003). These phenotypes are enhanced in the double mutant showing that BP-PNY have partially overlapping functions in specification of boundaries during internode growth (Smith and Hake, 2003). Genetic and transcriptome studies indicate that PNY modulates the activity of plant cell wall modifying enzymes required in loosening cell walls to allow organ initiation and internode elongation (Peaucelle et al., 2011; Etchells et al., 2012). BP regulates an overlapping set of genes and prevents premature deposition of lignin in elongating stems by direct repression of genes in the lignin biosynthetic pathway (Mele et al., 2003; Wang et al., 2006).

Genetic and expression studies show that *bp* and *pny* inflorescence defects are caused by the localized misexpression of lateral organ boundary genes *KNAT6, ATH1*, *BOP1/2* and to a lesser extent *KNAT2* in stems (Ragni et al., 2008; Khan et al., 2012a,b). Inactivation of *BOP1/2* or *KNAT6* or *ATH1* fully rescues *pny* defects to restore wild type inflorescence architecture. Similarly, inactivation of *BOP1/2* or *KNAT6* in combination with *KNAT2* or *ATH1* rescues *bp* defects in internode elongation and pedicel orientation. The regular pattern of vascular bundles and the pattern of lignin deposition in stems during secondary growth are reestablished in these mutants (Khan et al., 2012a,b). BOP1/2 require the functions of these downstream genes to exert changes in inflorescence architecture suggesting a linear pathway (Khan et al., 2012a,b). Further analysis of this module shows that BOP1 directly activates *ATH1* whereas activation of *KNAT6* is indirect (Khan et al., 2015). BP/STM are recently shown to promote xylem differentiation in the cambium through the repression of *BOP1* and *BOP2* (Liebsch et al., 2014). Thus, restriction of the *BOP1/2-ATH1-KNAT6* boundary module by BP-PNY is critical for plant architecture. Recent data reveal that BP directly represses *KNAT2* and *KNAT6* expression by recruiting the chromatin remodeling ATPase BRAHMA to the promoter (Zhao et al., 2015).

## Flower Initiation And Patterning

Floral inductive signals acting on the SAM cause restructuring to form the IM. Completion of this process requires the PNY and PNF BELL members. In *pny pnf* mutants, apices support the production of leaves, but internode elongation and flower initiation are blocked (Smith et al., 2004; Kanrar et al., 2008; Lal et al., 2011). Recent data show that this block is due to misexpression of BOP1/2 and its downstream effectors KNAT6 and ATH1 which prevent accumulation of floral meristem identity genes including *LFY*, *CAULIFLOWER* (*CAL*), and *APETALA1* (*AP1*) required for flower production (Khan et al., 2015). PNY in this network directly represses *BOP1/2* to maintain its expression at boundaries. One study shows that ectopic *BOP1/2* expression reduces responsiveness to FT by lowering the abundance of its binding partner FD (Andrés et al., 2015). Transcript profiling of *BOP1* overexpressing plants further identifies promotion of JA as a potential mechanism for inhibiting accumulation of SQUAMOSA PROMOTER BINDING-LIKE PROTEINS and counteracting responsiveness to GAs (Khan et al., 2015). Thus, the setting of lateral boundaries by PNY and PNF via the restriction of *BOP1/2-ATH1-KNAT6* expression is critical for meristem integrity and specification of flowers.

The floral meristem constitutes an AM whose rapid proliferation represses outgrowth of the subtending leaf (Long and Barton, 2000). Initiation of flowers is an auxin-dependent process similar to that in leaves (**Figure 1C**). Mutations in *MP* or *PIN1* result in naked IM “pins” lacking flowers due to misexpression of meristem/organ/boundary markers including *STM*, *LFY*, *CUC*, and *ANT* throughout the peripheral zone (Vernoux et al., 2000; Hay et al., 2006; Schuetz et al., 2008). MP integrates auxin and floral signals by directly activating *ANT*/*AIL6* which triggers proliferation in combination with *LFY* which activates flower development (Yamaguchi et al., 2013). Interestingly, MP does not bind to the *LFY* promoter during the vegetative stage indicating that binding is stage-specific (Yamaguchi et al., 2013). LFY reinforces this loop via direct activation of genes in the auxin pathway, direct activation of *RAX1*, and direct activation of *AP1* and *CAL* whose products confer floral fate (Wagner et al., 1999; William et al., 2004; Winter et al., 2011; Yamaguchi et al., 2013).

*BOP1/2* and *UNUSUAL FLORAL ORGANS* (*UFO*) are boundary regulators that facilitate LFY function. Genetic studies reveal that BOPs play a supporting role in the promotion of *LFY* expression (Karim et al., 2009), proliferation of the floral meristem, and determinacy in part through direct activation of *AP1* (Xu et al., 2010). Several of these functions are shared with UFO (Norberg et al., 2005; Xu et al., 2010; Risseeuw et al., 2013). Outgrowth of the floral meristem is delayed in *bop1 bop2*, *ufo-1*, and *lfy* mutants or absent in *bop1 bop2 lfy-1* triple mutants resulting in barren axils (Levin and Meyerowitz, 1995; Wilkinson and Haughn, 1995; Norberg et al., 2005; Xu et al., 2010). Inactivation of *BOP1/2* or *UFO CAL* greatly enhances the floral branching defect in *ap1* mutants caused by derepression of CK biosynthesis in sepal axils leading to ectopic FM initiation and loss of shoot determinacy (Levin and Meyerowitz, 1995; Xu et al., 2010; Han et al., 2014). UFO is the F-box subunit of an SCF-based E3 ubiquitin ligase complex which binds to LFY and functions as a transcriptional co-activator (Lee et al., 1997; Chae et al., 2008). Paradoxically, UFO stimulates LFY activity by directing ubiquitination of its transcriptional activation domain thus marking the protein for turnover which is required for maximal induction of target genes (Chae et al., 2008). Similar functions are shown for BOP and UFO orthologs in a variety of species (Khan et al., 2014; Vlad et al., 2014).

*Arabidopsis* flowers are composed of sepals, petals, stamens, and carpels arranged in four concentric whorls. LFY is responsible for this patterning by activating three sets of homeotic genes that function combinatorially according to the ABC model (Lohmann and Weigel, 2002). In *ufo* mutants, petals and stamens are reduced or absent and organs are fused or chimeric indicating disrupted boundaries in the flower (Levin and Meyerowitz, 1995; Wilkinson and Haughn, 1995). In *bop1 bop2* mutants, sepal-to-petal conversions and sepal-whorl organ fusions are localized to the abaxial side of flowers where *BOP1/2* are transcribed during late stage 2 (Hepworth et al., 2005; Xu et al., 2010). Inactivation of *BOP1/2* or *UFO* in a weak *lfy* background creates a strong *lfy* phenotype indicating closely related functions for these genes (Levin and Meyerowitz, 1995; Wilkinson and Haughn, 1995; Xu et al., 2010). *UFO* is activated in the dome of stage 2 flowers and resolves to a cup-shaped domain around *STM*-expressing cells in the central zone of stage 3 flowers (Lee et al., 1997; Samach et al., 1999) and is possibly involved in creating a boundary. Embryo expression of *UFO* is dependent on STM (Long and Barton, 1998) but the mutant has no obvious defects during this stage due to genetic redundancy.

Boundaries in the flower are maintained by various other boundary genes and stage-specific factors including the zinc-finger repressors RABBIT EARS (RBE; Takeda et al., 2004), and SUPERMAN (SUP; Sakai et al., 1995; Nibau et al., 2011) and HANABA TARANU, a GATA3-type transcriptional repressor (Zhao et al., 2004; Nawy et al., 2010). *CUC* genes are expressed between organ primordia and at the edges of whorls where they repress growth required to separate the floral organs and maintain boundaries between whorls (Ishida et al., 2000; Takada et al., 2001). *ATH1* controls basal floral organ boundaries and functions downstream of *CUC* genes (Gómez-Mena and Sablowski, 2008). *LOF1* (Gomez et al., 2011), *OBO1/LSH3*, and *OBO4/LSH4* (Cho and Zambryski, 2011) functioning downstream of *CUC1* are likely to contribute based on their expression patterns or overexpression phenotypes in the flower. Floral boundary defects are also observed in *bop1 bop2* and *jlo* flowers (Hepworth et al., 2005; Rast and Simon, 2012). Analogous to leaves, AS1–AS2, and JAG repress boundary genes including *CUC1* and *CUC2* to promote sepal and petal development (Xu et al., 2008).

PETAL LOSS (PTL) encodes a stage-specific trihelix transcription factor that represses growth in inter-sepal boundaries. In *ptl* mutants, petals are often absent, or show changes in shape, polarity, and fusion with sepals (Griffith et al., 1999). Sepal fusion is increased in *ptl cuc* mutants consistent with analysis showing that CUCs suppress upward growth of inter-sepal tissue at the boundary whereas PTL limits overgrowth of the inter-sepal zone required for petal initiation (Lampugnani et al., 2012, 2013). Petal initiation is highly sensitive to perturbations in growth and auxin distribution because primordia arise from 2 to 3 founder cells located in close proximity to the inter-sepal boundary (Lampugnani et al., 2012, 2013).

## Fruit Patterning And Dehiscence

**FIGURE 3:**
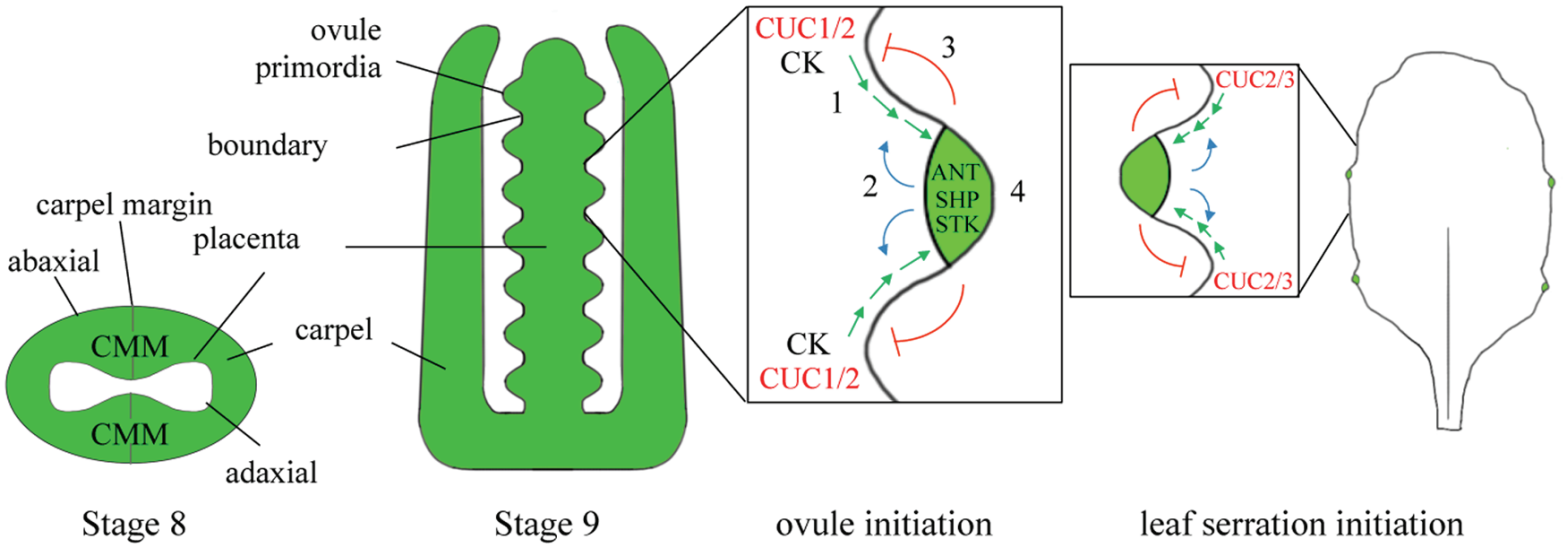
**Homologous networks for ovule initiation by the carpel marginal meristem (CMM) and patterning of leaf margin.** Two fused carpels in the center of the flower form a tubular structure that constitutes the gynoecium (stages 8 and 9). The inner fused surfaces of the carpels (equivalent to an adaxial leaf surface) form a ridge of meristematic tissue called the CMM that gives rise to ovules and septum. Ovules are initiated in a process that resembles creation of serrations on the leaf margin where CUC2 is required for leaf serration and CUC3 promotes serration growth (see text). (1) CUC1/2 in the placenta together with CK promote the formation of an auxin maximum. (2) Auxin positive feedback reinforces flow of auxin to the primordia tip. (3) Auxin negative feedback restricts CUC1/2 expression to the base of the ovule. (4) Once auxin reaches threshold levels, it switches on *ANT* which promotes outgrowth of the shoot and MADS box genes *SHP1/2* and *STK* which confer ovule identity. CUC2/3 expression overlaps between ovule primordia and is required for ovule separation (not shown). Adapted from (Cucinotta et al., 2014). Red lettering, SAM-leaf boundary genes.

**FIGURE 4:**
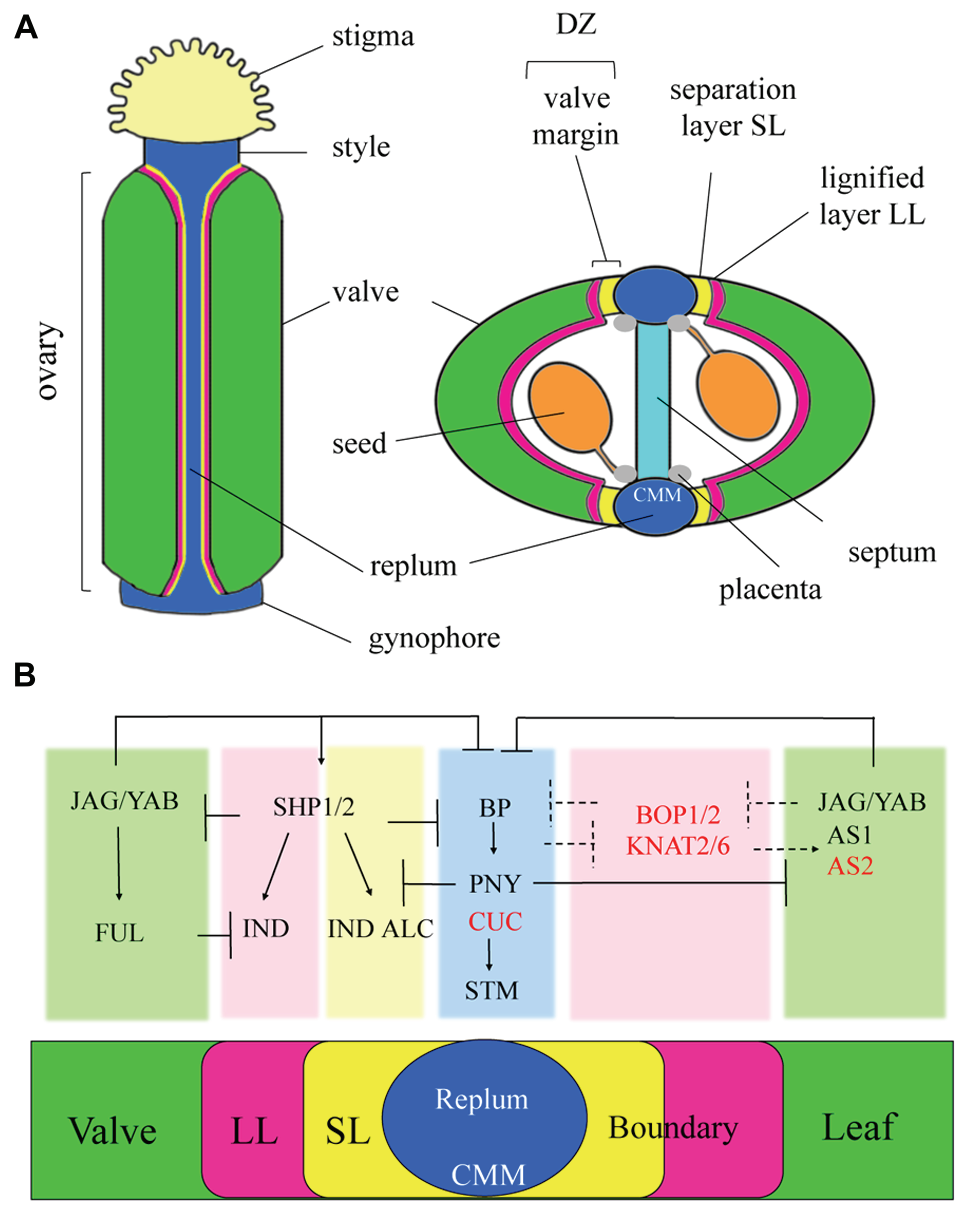
**Schematic of an *Arabidopsis* fruit and summary of networks for fruit patterning compared to the SAM-leaf boundary. (A)** The *Arabidopsis* fruit derived from two fused carpel valves that represent modified leaves. Valve margins are a lateral organ boundary specialized for dehiscence that joins the carpels to a meristematic tissue called the replum. The internal surface of the replum or CMM provides septum and placenta that gives rise to ovules that develop into seeds at fertilization. Differentiation of the valve margins requires GA, CK, and depletion of auxin (see text). When the fruit is mature, the valve margin differentiates to form the dehiscence zone (DZ) comprising two layers: a separation layer (SL) where the fruit will open and a lignified layer (LL) continuous with the lining of the fruit that provides tension required in spring-like opening of the fruit for seed dispersal. **(B)** Similar to their role in leaves, JAG and YAB factors together with AS1–AS2 are required in restricting expression of meristematic genes *BP* and *PNY* to the central replum domain and in restricting valve margin identity genes to the boundary junction. FUL is a stage-specific factor that confers valve identity and functions similarly to AS1–AS2 and JAB/YAB to correctly position the replum and valve margin identity domains. Red lettering, SAM-leaf boundary genes: CUC1/2 activate *STM* required in formation of the CMM and *BOP1/2* and *KNAT2/6* expressed in the valve margin of fruit are predicted to antagonize BP-PNY activity in the replum. Dashed arrows, hypothetical interactions.

The *Arabidopsis* fruit is derived from the gynoecium, which consists of two fused carpels representing modified leaves (Ferrándiz et al., 2010). The carpels (termed valves after fertilization) are joined to a central replum whose internal surface or carpel margin meristem provides ovules and a septum with transmitting tract. Valve margins are a specialized lateral organ boundary that forms at the valve/replum interface. They ensure the release of the seeds. Thus, three patterning elements define the transverse axis of the mature fruit: valves, valve margins, and replum. Many interactions defining the SAM-leaf boundary are conserved in ovule and fruit development (Ferrándiz et al., 2010; Reyes-Olalde et al., 2013; Arnaud and Pautot, 2014) and (**Figures 3** and **4**).

The carpel marginal meristem (CMM) forms internally at the junction between fused carpels, which is homologous to the marginal meristem of leaves (Pautot et al., 2001). The CMM produces two outgrowths fused centrally to form the septum and is flanked on both sides by the placenta that gives rise to ovules. *CUC* genes promote fusion of the carpel margins and CMM initiation via the activation of *STM* similar to their role in SAM (Kamiuchi et al., 2014). Defects in either of these genes impair placental function leading to a reduction in ovules (Endrizzi et al., 1996; Ishida et al., 2000; Scofield et al., 2007). CK also plays an important role in promoting meristematic activity. Visualization of CK using the synthetic TCS::GFP reporter shows CK in the CMM of young gynoecia. Increased levels of CK enhance replum size while decreased levels reduce replum size (Marsch-Martinez et al., 2012). Following establishment of the CMM, *CUC1/2* transcripts are detected in the placenta where together with CK they control the localization of PIN1 transporters in creating auxin maxima required for ovule initiation (Bencivenga et al., 2012; Galbiati et al., 2013; Cucinotta et al., 2014). In mutants that overexpress CK, the number of ovules increases (Bartrina et al., 2011). Conversely, where there is a reduction in CK response or defects in auxin synthesis, transport or signaling, the number of ovules decreases (Galbiati et al., 2013). This process is comparable to formation of serrations on the leaf margin (Bilsborough et al., 2011). Once an ovule is initiated, auxin and BR converge to activate *ANT* for proliferation of the ovule primordia (Galbiati et al., 2013; Huang et al., 2013). Auxin accumulation in the distal tip of the ovule ultimately restricts *CUC1/2* expression to boundaries in the ovule (Ishida et al., 2000). *CUC3* and *CUC2* are later expressed between ovule primordia overlapping in a few cells where they are redundantly required for ovule separation (Goncalves et al., 2015). *Cuc3* single mutants show rare fused ovules with defects more severe in the *cuc3 cuc2* double mutant. LOF1 may also play a role based on its expression pattern in the inner medial ridges, the placenta, and at the base of the ovules marking these domains as lateral organ boundaries (Gomez et al., 2011). MADS-box transcription factors AGAMOUS, SHATTERPROOF1/2 (SHP1/2), and SEEDSTICK (STK) confer ovule identity (Ishida et al., 2000; Pinyopich et al., 2003; Galbiati et al., 2013).

After fertilization, the ovules develop into seeds and the fruit enlarges. At maturity, the valve margins undergo secondary differentiation to form the dehiscence zone where the fruit opens. The dehiscence zone has two cell layers: a separation layer adjacent to the replum and a lignified layer. The separation layer produces enzymes that break down the middle lamella, a layer of pectin that cements cell walls together. The lignified layer is continuous with the inner layer of the fruit and is required for spring-like opening of the fruit (Ferrándiz et al., 2010).

The valve margin expresses a pair of stage-specific MADS-box transcription factors encoded by *SHP1/2* and boundary genes *KNAT2/6* and *BOP1/2* that are activated later during carpel development (Liljegren et al., 2000; Ragni et al., 2008; Khan et al., 2012b). SHP1/2 confer valve margin identity via activation of downstream bHLH transcription factors INDEHISCENT (IND) and ALCATRAZ (ALC) (Liljegren et al., 2004). IND is required for differentiation of lignified and separation layers of the valve margin whereas ALC/SPATULA (SPT) are required for differentiation of the separation layer (Rajani and Sundaresan, 2001; Liljegren et al., 2004; Girin et al., 2011; Groszmann et al., 2011). IND leads to the depletion of auxin in valve margins by relocating PIN1 transporters (Sorefan et al., 2009). IND also promotes GA production, which releases ALC and SPT proteins from DELLA repression allowing formation of a productive complex to specify the separation layer (Arnaud et al., 2010). The auxin and GA pathways seem to be independent, since the auxin minimum is maintained in GA deficient mutants. Visualization of CK in mature gynoecium shows CK in valve margins, and this localization depends on IND and SHP1/2 activity (Marsch-Martinez et al., 2012). Interestingly, CK restores valve margins in *shp1 shp2* and *ind* mutants indicating that CK functions downstream of these regulators. In contrast, a complementary pattern is observed for auxin with a synthetic DR5 reporter detected only in replum and valves. Thus, CK promotes valve margins. CK may contribute to the depletion of auxin in valve margins via the localization of PIN transporters (Marsch-Martinez et al., 2012) but it is unknown if the depletion of auxin is required for the accumulation of CK in valve boundaries or if the valve margin regulators IND and SHP1/2 activate this pathway.

The formation and relative size of external domains in the fruit: valves, valve margin, and replum are governed by antagonistic interactions analogous to those at the leaf-boundary-SAM interface (González-Reig et al., 2012). JAG and YABBY (YAB) members FILAMENTOUS (FIL)/YAB3 also found in leaves activate the MADS-box gene *FRUITFULL* (*FUL*), which is required for elongation and differentiation of the valves, and *SHP1/2*, which confer valve-margin identity (Dinneny et al., 2005). FUL in turn represses *SHP/IND/ALC* to set the valve margin boundary. Fruits in a *ful* mutant are constricted and ectopically lignified due to the misexpression of valve margin genes (Ferrándiz et al., 2000; Liljegren et al., 2004). AS1–AS2 and JAG/FIL/YAB3 reprise their roles in the leaf by restricting *BP* expression to the replum. In *as1* or *as2* mutants or in *jag fil yab3* triple mutants, valve width is reduced and the replum is expanded due to an increase in *BP* expression (Alonso-Cantabrana et al., 2007; González-Reig et al., 2012). BP, which interacts with PNY, also known as REPLUMLESS (Roeder et al., 2003), activates its expression, and contributes redundantly with PNY in maintaining the replum in part by repressing valve and valve-margin identity genes (Alonso-Cantabrana et al., 2007). Thus, inactivation of *JAG/FIL/YAB3* or *SHP/IND* genes partially rescues replum formation in a *pny* mutant (Roeder et al., 2003; Dinneny et al., 2005; Alonso-Cantabrana et al., 2007). Other factors identified are APETALA2 which prevents overgrowth of the replum and valve margin by repressing *BP/PNY* and valve margin identity genes (Ripoll et al., 2011) and the zinc finger transcription factor NO TRANSMITTING TRACT which promotes replum development by activating BP (Marsch-Martinez et al., 2014).

The role of boundary genes in fruit patterning and dehiscence is worth exploring. Mutations in *SHP1/2* or the different stage-specific *bHLH* genes block dehiscence (Ferrándiz et al., 2010) but this is not case for *KNAT2/6* or *BOP1/2*. Nevertheless, inactivation of these genes restores replum formation in *pny* mutants showing that the antagonistic interaction between BOP1/2- KNAT6/2 and PNY also control fruit patterning (Ragni et al., 2008; Khan et al., 2012b). Based on the role of this module in other boundary contexts, BOP1/2 and KNAT2/6 likely contribute to repression of *BP/PNY* to control replum size, specialization of cells in the separation layer, and formation of lignified cell layers (McKim et al., 2008; Khan et al., 2012a,b). Similar interactions among meristem and boundary genes regulate abscission.

## Abscission

**FIGURE 5:**
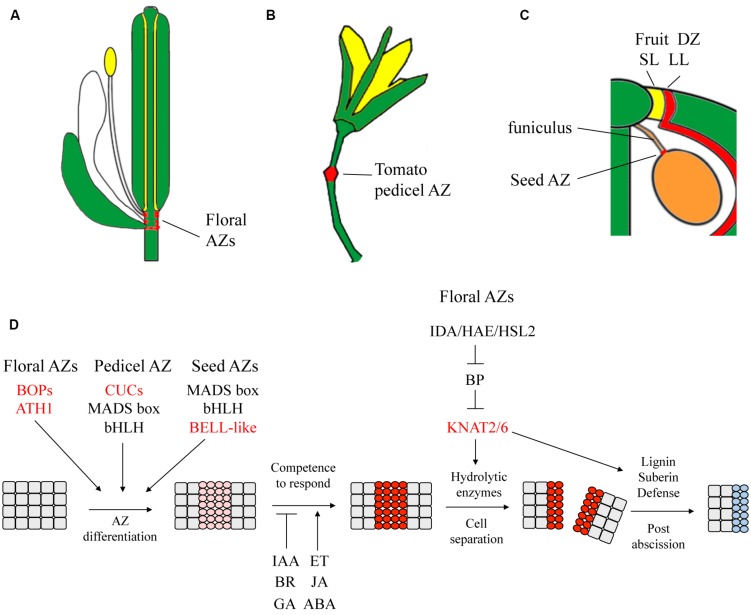
**Summary of pathways that control the development of abscission zones (AZs).** Placement of AZs (in red) are shown for **(A)** *Arabidopsis* flower, **(B)** tomato pedicel, and **(C)** *Arabidopsis* seed and dehiscence zone. **(D)** The current accepted model for abscission defines four key stages: differentiation of AZ cells (pink), AZ cells acquire competence to respond to hormone inductive signals (red), activation of abscission and detachment (red), and differentiation of a protective layer over the scar (blue). Red lettering, SAM-leaf boundary genes. Dashed lines, hypothetical pathway. IAA, auxin; BR, brassinosteroid; GA, gibberellin; ET, ethylene; CK, cytokinin; JA, jasmonic acid. Adapted from (Leibfried et al., 2005; Niederhuth et al., 2013).

Abscission zones (AZs) are typically located at lateral organ boundaries in the plant at the base of leaves, floral organs, or seeds [(Estornell et al., 2013) and **Figure 5**]. In *Arabidopsis*, AZs comprised of small, densely cytoplasmic cells form simultaneously with the boundary for detachment of floral organs and seeds (McKim et al., 2008). At anthesis, AZ cells acquire competence to respond to abscission signals and secrete cell-wall modifying and hydrolyzing enzymes that degrade the middle lamella between two adjacent cell files. Ethylene, JA, and abscisic acid are promotive signals for abscission whereas auxin, GA, and BRs are inhibitory (for reviews: Estornell et al., 2013; Niederhuth et al., 2013; Kim, 2014). Depletion of auxin from the AZ is shown to improve sensitivity to ethylene in controlling the timing of abscission (Estornell et al., 2013) reminiscent of other boundaries.

Boundary genes are required for both differentiation and separation phases of abscission. A variety of plant species lacking BOP activity fail to form an AZ (McKim et al., 2008; Wu et al., 2012; Couzigou et al., 2015). BOP1/2 quite possibly perform this function via ATH1 and KNAT2/KNAT6. Inactivation of *ATH1* has a mild abscission defect in which formation of the stamen AZs is delayed. A functional AZ eventually develops and organs detach (Gómez-Mena and Sablowski, 2008). Reprising its role in leaves, AS1 positions the medial sepal and petal AZs in *Arabidopsis* via restriction of BP activity, which in turn restricts expression of the HAESA receptor-like kinase (Gubert et al., 2014). This was discovered through isolation of a new allele, *as1-22*, which shows a delayed abscission defect (Gubert et al., 2014).

The activation of floral organ abscission involves the peptide INFLORESCENCE DEFICIENT IN ABSCISSION (IDA) and the receptor like kinase HAESA and HAESA-LIKE2 (HAE-HSL2) signaling pathway. Low levels of *IDA* and *HAESA* transcripts are also expressed in the mature dehiscence zones of the fruit (Stenvik et al., 2008). Activation of cell separation by IDA-HAE/HSL2 signaling antagonizes BP activity leading to up-regulation of *KNAT2* and *KNAT6* and accumulation of cell-wall modification and hydrolytic enzymes that mediate separation (Shi et al., 2011). Waxes, suberin, lignin, and pathogenesis-related genes are also induced in protecting exposed cells from dehydration and pathogen attack (Estornell et al., 2013; Kim, 2014). Plants overexpressing *BOP1* show significant enrichment for genes involved in lignin biosynthesis, stress and pathogen resistance (Khan et al., 2012a, 2015) suggesting a potential role in post-abscission events at the boundary.

Not all AZs form at a lateral organ boundary (Estornell et al., 2013). The pedicel AZ in tomato is a well-studied example in which a small groove in the floral pedicel leads to differentiation of a “joint” where abscission takes place. Two MADS box proteins JOINTLESS and MACROCALYX form a complex that regulates formation of the pedicel AZ together with LAS required in AM production (Schumacher et al., 1999; Nakano et al., 2012). CUC2 homolog GOB has been proposed to be involved in the regulation of the onset of abscission based on its expression in tomato pedicel AZs (Nakano et al., 2013). Tomato *KNAT6* (*TKN3*), *KNAT2* (*TKN4*), and the *KNATM* homolog *KD1* are all highly expressed in pedicel AZs similar to *Arabidopsis*. Silencing of *KD1* delays abscission by increasing auxin content and overexpression of *KD1* has the opposite effect (Ma et al., 2015). Hormonal control and transcript profiling of activated AZs in *Arabidopsis* and tomato are very similar despite these apparent differences (Estornell et al., 2013; Ito and Nakano, 2015).

An AZ at the base of the seed allows detachment from the funiculus. In *Arabidopsis*, specification of this AZ requires the MADS-box protein SEEDSTICK and the bHLH factor HECATE3 (Pinyopich et al., 2003; Ogawa et al., 2009). BOP1/2 do not seem to be expressed in the ovule nor is there an obvious requirement for the IDA-HAE/HLS2 signaling pathway (Estornell et al., 2013). This suggests a greater alignment with processes that control dehiscence. The role of PNY has yet to be investigated in seed dehiscence. Interestingly, domesticated japonica rice is selected for a promoter mutation in the *PNY/RPL* homolog *qSHATTERING1* that depletes expression from the abscission layer to inhibit seed shatter (Konishi et al., 2006). *qSH1* and a related gene *SH5* are required for development of the pedicel AZ and inhibition of these genes reduces shatter by promoting lignin biosynthesis (Yoon et al., 2014). Overexpression of wheat *TaqSH1* in *Arabidopsis* delays abscission and down regulates abscission-promoting genes suggesting that TaqSH1 can function as an upstream regulator of the *IDA-HAESA-KNAT* pathway (Zhang et al., 2013). Remarkably, the same mutation in *qSH1* was found in *Brassica rapa* which exhibit a narrow replum compared to *Arabidopsis* (Arnaud et al., 2011). Collectively, these data show remarkable overlap between separation processes involved in pod shatter, floral organ abscission, and seed dehiscence in which lateral organ boundary genes play a key role.

## Concluding Remarks

In this review, we illustrate the importance of boundaries throughout development. Studies have revealed a number of genes including *CUCs*, *TALEs*, and *BOP* that play a recurring role thoughout the life cycle. During the reproductive phase, their activities are embedded within specialized networks required for inflorescence, flower, and fruit development. How these pathways are integrated is only partly understood. Many questions also remain concerning the role of these genes in the SAM. Molecular links between *CUC* genes that confer boundary identity and BOP-TALE factors are not well-established. Recent studies in monocots have shed light on the role of KNOX transcription factors in initiating boundaries but application of this model to dicots is not yet confirmed. While significant progress has been made in understanding how KNOX factors regulate hormone abundance, such links are still largely missing for CUC and BOP factors at the boundary. These factors also repress growth and cell division but few targets have been identified to date and their hierarchy is unclear%. Identification of transcriptional targets is key to understanding how these factors pattern the boundary. Understanding how these networks translate to boundaries during reproductive development is still in its infancy. In particular, the influence of BR and the role of JLO is not yet explored. The function of BOP and TALE factors in fruit and in abscission is also unclear. Finally, the contribution of these networks to the activity of lateral meristems responsible for secondary growth in stems and roots is only partly understood. Transcriptome analysis and further exploration of these factors in boundaries in other species will establish the extent to which the mode of action of these factors is conserved in development.

Wolfgang von Goethe in 1790 first proposed that plants are formed from repeating units called phytomers (Bossinger, 2009). As developmental contexts are further explored this notion can be extended to the molecular level. Work in the last decade in a variety of species makes it obvious that simple patterns established with formation of the embryonic plant underlie pattern development at all stages of plant development. As such, there is much to be learned from drawing parallels and insights from different developmental contexts. Lateral organ boundaries are a particularly intriguing part of the puzzle as they regulate the balance between meristem activity and growth and represent a fundamental gatekeeper of meristem function in plants.

## Conflict of Interest Statement

The authors declare that the research was conducted in the absence of any commercial or financial relationships that could be construed as a potential conflict of interest.
